# Comparison of relationships among French adult siblings with or without schizophrenia using the ASRQ-S: mediating effect on emotional distress

**DOI:** 10.1186/s12888-020-02510-6

**Published:** 2020-03-13

**Authors:** Léa Plessis, Hélène Wilquin, Jean-Baptiste Pavani, Evelyne Bouteyre

**Affiliations:** 1 LPCPP, Aix Marseille University, Aix-en-Provence, France; 2 PsyCLE, Aix Marseille University, Aix-en-Provence, France

**Keywords:** Sibling relationships, Schizophrenia, Matched samples, Emotional distress

## Abstract

**Background:**

Good sibling relationships in adulthood are known to be a protective factor for mental health. The present study examined and compared the relationships of siblings with either a healthy brother or sister or one experiencing schizophrenia.

**Methods:**

In the first phase, we ran a statistical comparison of the two sibling groups on the quality of their sibling relationships (warmth, conflict, and rivalry), emotional distress, and self-esteem. In the second phase, we looked at whether the quality of the sibling relationship modifies the impact of having a brother or sister with schizophrenia on emotional distress and self-esteem.

**Results:**

Results showed that sibling relationships in schizophrenia are less warm and are characterized by heightened rivalry and conflict. In addition, analysis revealed a mediating effect of sibling relationship on the emotional distress of siblings with a brother/sister diagnosed with schizophrenia.

**Conclusion:**

More needs to be done to enhance the mental health of adults who have a brother or sister with schizophrenia, notably via their sibling relationships.

## Background

Schizophrenia (SZ) affects almost 1% of the population and has severe consequences for both the patient and the patient’s family.

Published research on the adult siblings of individuals with SZ has so far been restricted to the impact of a first episode of psychosis (FEP) on siblings in their early 20s [1–4], their adaptation strategies [5–8], and the extent to which they fulfil the role of informal caregiver [9–14]. To our knowledge, healthy adults’ perceptions of their relationship with a brother or sister experiencing SZ have never been investigated beyond this first episode. We therefore carried out a novel comparison between the adult siblings of individuals with SZ and matched sets of siblings drawn from the general population on their experience of sibling relationships.

### Sibling relationships

A subsystem of the family system, the sibling relationship is classically studied according to three dimensions: warmth, conflict, and rivalry [15, 16]. *Warmth* can be defined as closeness and support within the relationship, while *conflict* refers to disagreements and quarrels within that relationship, and *rivalry* corresponds to competition for parental affection and attention.

Good sibling relationships can be highly beneficial in terms of cognitive [17, 18], emotional [19, 20] and social development [21, 22]. High on warmth and low on both conflict and rivalry [23], they are a prime protective factor for mental health [24], linked to greater self-confidence, better social skills, and general wellbeing [23, 25, 26]. They can also shield individuals from loneliness [15, 27, 28] and keep depression and anxiety at bay [15, 29].

When individuals reach adulthood, life events such as finishing their formal education, entering the world of work, meeting new people, marrying, and starting a family often lead them to pursue fresh aspirations beyond the family circle [30, 31]. Unsurprisingly, most studies show that all three dimensions of sibling relationships are expressed less intensely in adulthood [32–35], although warmth prevails over both conflict and rivalry [21, 33, 36, 37]. Research on sibling relationships in adulthood is nevertheless particularly relevant, as these are the longest relationships that individuals have in their lifetime, outlasting those between parents and children or between spouses [21, 38, 39].

### Sibling relationships in adulthood and disability

When individuals experience disability, they continue to receive support from their siblings in adulthood, although it may take several different forms (e.g., emotional or financial). Healthy individuals who assist and keep in regular contact with a brother or sister who has a disability report more positive feelings about their general wellbeing and their sibling relationships [40, 41].

Depending on the disability, some symptoms may nonetheless adversely affect relations with an ill sibling. For example, the siblings of individuals with autism report poorer relationships than the siblings of individuals with Down syndrome [42]. According to Travis and Sigman [43], this can probably be attributed to the particular nature of autism symptoms, which are characterized by a social deficit and limited interpersonal relations (impaired communication, social cognition, emotions and social relations).

Intellectual disability and developmental disorders are chronic in nature, whereas mental disorders bring with them the risk of relapse and variable symptoms, and may have more episodic manifestations. SZ usually manifests itself for the first time between the ages of 15 and 25 years, whereas autism is generally diagnosed around the age of 3 years [44]. Adaptation should not, however, be equated with acceptance, let alone understanding, of the illness, which can require a considerable length of time [45, 46].

### Sibling relationships in SZ

When an individual is diagnosed with a mental disorder such as SZ, it can come as a huge emotional shock to his or her healthy siblings, who typically experience a mixture of stress, denial, despair, fear, guilt and impotence [3, 4]. The severity of the symptoms (e.g., delusions, hallucinations, behavioral problems, anhedonia, cognitive disorders and social withdrawal), combined with the chronic nature of the illness, places a heavy daily burden on all family members [47]. Moreover, as SZ often appears during adolescence or early adulthood, the ill person’s siblings are generally still living at home. In this context, Bowman et al. [1] and Sin et al. [3, 4] studied the impact of an FEP on sibling relationships, but without a control group (e.g. siblings without a brother/sister with SZ). More specifically, Bowman et al. used the Adult Sibling Relationship Questionnaire (ASRQ) to examine how healthy individuals (*N* = 157; *M*_age_ = 21.7 years) with a brother or sister who had experienced an FEP within the previous 18 months perceived their sibling relationships [1]. Results suggested that a history of violent behavior by the ill brother or sister was predictive of poorer sibling relations (less warmth, more conflict, and more rivalry).

## The present study

The present study had two objectives. The first was to compare adult siblings with or without a brother or sister experiencing SZ on the quality of their sibling relationships, emotional distress, and self-esteem. Based on the literature, we expected the siblings of individuals with SZ to report less warmth, more conflict, and more rivalry than siblings drawn from the general population. The second objective was to clarify how the quality of the sibling relationship modifies the impact of having a brother or sister with SZ on emotional distress and self-esteem. We postulated that the quality of the sibling relationship has a mediating effect on both the emotional distress and self-esteem of siblings with a brother or sister diagnosed with SZ.

## Methods

### Participants

#### Two initial samples

We recruited two initial samples of French volunteers: a large sample of 1444 siblings (76.5% female; *M*_age_ = 25.91 years, *SD* = 8.31) drawn from the general population between September and November 2017; and a smaller sample of 201 siblings (77.1% female; *M*_age_ = 37.9 years, *SD* = 12.08) with a brother or sister with SZ between November 2017 and February 2018. All participants (*N* = 1645) completed an online questionnaire. The inclusion criteria for all participants were 1) aged at least 18 years, and 2) at least one sibling aged 18 years or over. For participants in the SZ sample, there was a third criterion: a sibling with SZ. Participants in this group reported having a sibling who had been diagnosed with SZ by a healthcare professional.

#### Sample matching procedure

Each participant in the SZ sibling sample was matched with a participant in the general population sample on four criteria: age (within 5 years), sex, sex of target sibling, and birth order (younger or older than target sibling).

#### Two matched groups

With these four criteria, we were able to match 187 participants in the SZ sibling sample with 187 siblings from the general population. We therefore ended up with two matched groups, each with 187 participants (*N* = 374). In each group, the mean age was 35.9 years (*SD* = 10.7), 46.5% of participants were younger than the target sibling, 49.7% were older, and 3.7% were twins. In both samples, 77.5% of the participants were women, most of whom responded about their relationship with a brother (80.7%). Fourteen participants in the original sample of 201 individuals with a sibling diagnosed with SZ could not be paired, mostly because of their age: nine of them were aged over 64 years, whereas the oldest participant in the general population sample was 64 years. The remaining five were twins for whom we were unable to find a matching twin of the same sex in the general sample (*N* = 1444).

### Procedures

Janghorban, Roudsari, and Taghipour recommended recruiting participants via social media, instead of relying solely on psychiatric institutions or charitable bodies, in order to reach a broader population [48]. Participants were therefore recruited via social media and through the Union Nationale de Familles ou Amis de Personnes Malades et/ou Handicapées Psychiques^1^ (UNAFAM), a French charity that offers support to the families of persons with chronic mental disorders.

An electronic link to an online form was sent by social media group administrators, organization websites and charity newsletters to potential participants. After reading an information letter, participants had to validate their consent online. They could then gain access to the questionnaire, a self-administered online questionnaire that took approximately 40 min to complete.

### Ethical considerations

The research protocol was approved by Lille University’s ethics committee for human-based research (2018–276-S61), and complied with the principles of the Declaration of Helsinki [49].

### Measures

#### Sociodemographic data

We collected participants’ sociodemographic data and information about their family structure (see Table 1).

**Table 1:** Participants’ sociodemographic and sibling-related characteristics

Variables	Modality (for categorical variables)	Total sample (*N* = 374)	General population group (*n* = 187)	Clinical siblings (*n* = 187)	*t* or chi^2^ values for unmatched variables
Sex (%)	Female	288 (77.00)	144 (77.00)	144 (77.00)	
Mean age in years (*SD*)		35.96 (10.75)	35.93 (10.76)	36 (10.77)	− 0.07
Professional activity (%)	None	90 (24.06)	55 (29.41)	35 (18.72)	5.28*
Sex of target brother or sister (%)	Female	74 (19.79)	37 (19.79)	37 (19.79)	
Mean age difference between the two siblings (*SD*)		−0.26 (5.24)	− 0.67 (4.29)	0.14 (6.02)	−1.49
Participant’s birth order (%)	Youngest	94 (25.13)	39 (20.86)	55 (29.41)	2.37
	Intermediate	131 (35.03)	67 (35.83)	64 (34.22)	0.05
	Oldest	149 (39.84)	81 (43.32)	68 (36.36)	1.12
Target brother or sister’s birth order (%)	Youngest	77 (20.59)	21 (11.23)	56 (29.9)	18.91***
	Intermediate	168 (44.92)	101 (54.01)	67 (35.83)	11.77***
	Oldest	129 (34.49)	65 (34.76)	64 (34.22)	0
Living with sibling (%)	Same	19 (4.95)	7 (3.74)	12 (6.42)	0.89

Note: *** *p* ≤ 0.001, ** *p* ≤ 0.01, * *p* ≤ 0.05

#### Adult sibling relationship questionnaire-short form (ASRQ-S)

The ASRQ-S is a self-report questionnaire assessing the qualitative features of sibling relationships in young adulthood and beyond. Participants were asked to report on a single sibling relationship. The original long-form version of the ASRQ (81 items) was developed by Stocker et al. [16] as an age-appropriate extension of the Sibling Relationship Questionnaire [50].

The short form of the ASRQ (ASRQ-S), developed by Lanthier, Stocker, and Furman but not yet validated or published, includes 47 of the 81 items in the full ASRQ [16] (cf. Supplementary Material). These 47 items are divided into eight subscales corresponding to the three above-mentioned factors: Knowledge, Intimacy, and Emotional Support (Warmth); Antagonism, Dominance, and Quarreling (Conflict); and Maternal Rivalry and Paternal Rivalry (Rivalry).

The ASRQ-S was translated into French using Vallerand’s back-translation procedure, after obtaining the consent of the original authors [51]. A native English bilingual translated the English version of the ARSQ-S into French, and a second bilingual translated this French version back into English. When compared, the two English versions were initially found to have substantial incongruities. The French version was therefore self-administered by 10 siblings to identify potential problems or ambiguities arising from the translation. Their responses were used to produce the final French version of the ARSQ-S.

Three of the items (Items 10, 11 and 27) making up the dominance subscale were deleted from the French version because the component coefficients were not conclusive (> .30). After deleting these items, the French version of the ASRQ-S contained 44 items shared between three main factors, which themselves were divided into eight subscales. The French version of the ASRQ-S shows good internal consistency (minimum α = 0.65, maximum α = 0.96).

#### Indicator of emotional distress

We created an emotional distress indicator, using the French version of the Hospital Anxiety and Depression (HAD) scale translated and validated by Lepine, Godchau, and Brun [52]. This scale has two dimensions: depressive symptomatology and anxious symptomatology. Each dimension includes seven items rated on a 4-point Likert-like scale ranging from *Never* to *Most of the time*. The psychometric qualities of the HAD scale have good internal consistency (α = .81–.90).

As anxiety and depression are very strongly correlated (*r* = 0.53), we summed the two scores to obtain the emotional distress indicator.

#### Self-esteem

Self-esteem was assessed with the French version of Rosenberg’s Self-Esteem Scale (SES). Translated and validated by Vallieres and Vallerand [53], the SES (e.g., “I would like to have more respect for myself” or “Sometimes I feel really useless”) includes 10 items evaluated on a Likert scale ranging from 1 (*Strongly disagree*) to 4 (*Strongly agree*). The psychometric qualities of the instrument are satisfactory (α = .70–.90).

### Data analysis strategy

**Fig. 1:**
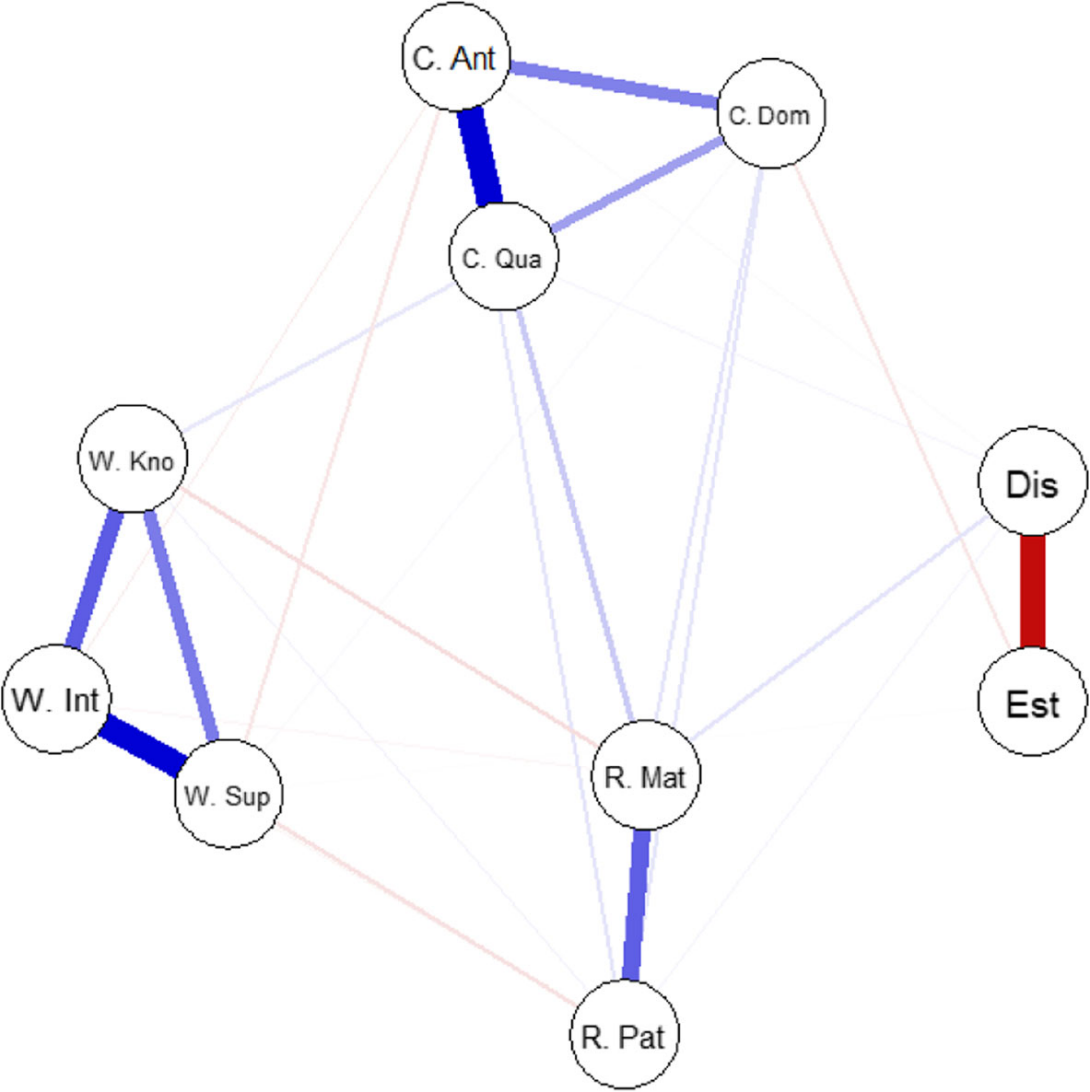
Network of variables of interest. Note: W. Kno = knowledge; W. Int = intimacy; W. Sup = support; C. Ant = antagonism; C. Dom = dominance; C. Qua = quarreling; R. Mat = maternal rivalry; R. Pat = paternal rivalry. The estimated network was a sparse Gaussian graphical model. The distances between the variables in the figure reflect the strength of their correlations

The data collected in the present study were analyzed using R [54]. Descriptive statistics of our 13 variables of interest obtained from the ASRQ-S, HAD scale and SES were computed with the two samples of siblings (Table 2). The correlations between these variables were also calculated. To facilitate the understanding of these correlations, they are displayed in both numerical (i.e., correlation matrix; see Table 2) and graphical (i.e., sparse Gaussian graphical model^2^; see Fig. 1) form. This model was computed using the qgraph R package [55].

**Table 2:** Means, standard deviations, and skewness coefficients of variables of interest for the whole sample (*N* = 374), and correlations between these variables

	1	2	3	4	5	6	7	8	9	10	11	12	13
1. Warmth													
2. W. Kno	0.90												
3. W. Int	0.94	0.77											
4. W. Sup	0.94	0.76	0.85										
5. Conflict	−.19	− 0.09	−0.22	− 0.21									
6. C. Ant	−.24	− 0.13	− 0.26	− 0.26	0.90								
7. C. Dom	−.12	− 0.07	− 0.15	− 0.10	0.83	0.62							
8. C. Qua	−0.14	− 0.05	− 0.16	− 0.17	0.90	0.77	0.59						
9. Rivalry	−0.31	−0.25	−0.29	− 0.30	0.37	0.32	0.31	0.35					
10. R. Mat	−0.27	− 0.24	− 0.25	− 0.25	0.35	0.29	0.29	0.34	0.88				
11. R. Pat	−0.23	−0.13	− 0.24	− 0.25	0.31	0.30	0.27	0.25	0.87	0.48			
12. Distress	−0.04	− 0.03	− 0.03	− 0.06	0.16	0.14	0.13	0.14	0.18	0.16	0.12		
13. Esteem	0.01	0.03	0.01	−0.01	−0.15	−0.11	− 0.15	−0.13	− 0.12	− 0.10	−0.09	− 0.60	
Mean	2.8	2.91	2.74	2.74	1.92	1.79	1.74	2.22	0.59	0.63	0.54	13.96	30.38
*SD*	0.91	0.87	0.98	1.07	0.72	0.74	0.83	0.88	0.52	0.57	0.59	5.96	6.47
Skewness	0.23	0.07	0.25	0.25	0.85	0.80	1.23	0.69	0.67	0.67	0.97	0.66	−0.40

*Note*. *SD* Standard deviation, *W. Kno* Knowledge, *W. Int* Intimacy, *W. Sup* Support, *C. Ant* Antagonism, *C. Dom* Dominance; *C. Qua* Quarreling, *R. Mat* Maternal rivalry, *R. Pat* Paternal rivalry. All correlations above 0.10 were statistically significant at the 0.05 level. Whereas the means, standard deviations, and skewness coefficients for dominance were computed on the raw variables, the correlations involving dominance were computed after the latter had been log transformed

Next, we ran means comparisons between the two samples (siblings from the general population vs. siblings with a brother or sister with SZ). Paired Student *t* tests were computed to determine whether the two samples had different means on our variables of interest (see Table 3). The subscales assessing maternal and paternal rivalry contained missing data, as participants had the option of not answering the rivalry items if one of their parents had died. Whenever data were missing, we performed the analyses on the remaining data.

**Table 3:** Means comparisons between siblings with a healthy brother or sister and siblings with a brother or sister experiencing schizophrenia on the quality of their sibling relationships, distress, and self-esteem

	General population group (*n* = 187)		Clinical siblings (*n* = 187)				
Variables	*M*	*SD*	*M*	*SD*	*t*	*p*	*d*
Warmth	3.07	0.94	2.53	0.78	−6.04	< 0.001***	−0.44
W. Know	3.1	0.87	2.72	0.82	−4.48	< 0.001***	−0.33
W. Int	2.99	1.04	2.5	0.87	−4.99	< 0.001***	−0.36
W. Sup	3.11	1.1	2.38	0.89	−7.03	< 0.001***	−0.51
Conflict	1.84	0.66	1.99	0.76	2.11	0.04*	0.15
C. Ant	1.7	0.72	1.88	0.76	2.48	0.01**	0.18
C. Dom	0.43	0.43	0.48	0.43	1.02	0.31	0.07
C. Qua	2.12	0.76	2.32	0.97	2.25	0.03*	0.16
Rivalry	0.46	0.45	0.73	0.55	4.99	< 0.001***	0.44
R. Mat	0.5	0.51	0.75	0.59	3.7	< 0.001***	0.33
R. Pat	0.38	0.49	0.7	0.64	4.74	< 0.001***	0.33
Distress	13.21	5.36	14.71	6.42	2.42	0.02*	0.18
Esteem	30.22	6.31	30.54	6.63	0.47	0.64	0.03

*Note*. *Ctrl* Siblings with a healthy brother or sister, *SZ* Siblings with a brother or sister experiencing schizophrenia, M Mean, *SD* Standard deviation, *W. Know* Knowledge, *W. Int* Intimacy, *W. Sup* Support, *C. Ant* Antagonism, *C. Dom* Dominance, *C. Qua* Quarreling, *R. Mat* Maternal rivalry, *R. Pat* Paternal rivalry

*** *p* ≤ 0.001. ** *p* ≤ 0.01. * *p* ≤ 0.05

Finally, we performed mediation analyses to determine whether the quality of the sibling relationship mediated the putative effect of having a sibling with SZ on distress and self-esteem. We used the mediation R package [56] and followed the four steps recommended by Baron and Kenny [57].

## Results

### Descriptive statistics

Descriptive statistics for the variables of interest in the present study are set out in Table 2 and Fig. 1. At this descriptive level, three main results emerged.

First, correlations between the eight subscales of the ASRQ-S supported its construct validity. For instance, Table 2 shows that the weakest correlation between two subscales (i.e., Maternal Rivalry and Paternal Rivalry) supposed to belong to the same dimension was equal to 0.48. This was still a strong correlation, and it was stronger than any correlation between two subscales supposed to belong to different dimensions. In the same vein, Fig. 1 shows that, as expected, the eight subscales of the ASRQ-S were organized into three separate clusters (i.e., warmth, conflict, and rivalry).

Second, conflict and rivalry appeared to be more closely correlated with emotional distress and self-esteem than warmth was. Thus, the three dimensions of sibling relationship quality were differently associated with wellbeing indicators.

Third, one variable (i.e., dominance) was positively skewed. The log-transformed version of this variable was therefore used in the analyses reported in the present study, including the correlations displayed in Table 2 and Fig. 1.

### Means comparisons

We tested the hypotheses that, compared with siblings who have healthy brothers and sisters, siblings who have a brother or sister with SZ display less warmth and more conflict and rivalry in their sibling relationships. They are also more distressed and have lower self-esteem.

Our two hypotheses were generally confirmed. Compared with controls, siblings who had a brother or sister with SZ rated their sibling relationships more poorly, and reported more emotional distress (see Table 3). We failed to find a statistically significant difference between the two groups on only two variables: dominance, *t*_(186)_ = 1.02, *p* = 0.31, *d* = 0.07, and self-esteem, *t*_(186)_ = 0.47, *p* = 0.64, *d* = 0.03.

### Mediation analyses

To establish the presence of mediating effects, we followed the four steps recommended by Baron and Kenny [57]. Results are shown in Table 4.

**Table 4:** Mediation analyses for the five sequences of variables meeting Baron and Kenny’s (1986) criteria for the presence of mediation effects

Predictor	Outcome	Mediator	*n*	Total effect	Mediated effect	*p*
Sz vs. Ctrl	Distress	Antagonism	374	0.25	0.03 (11%)	0.032
Sz vs. Ctrl	Distress	Quarrel	374	0.25	0.03 (10%)	0.049
Sz vs. Ctrl	Distress	Rivalry	374	0.21	0.09 (39%)	0.002
Sz vs. Ctrl	Distress	Maternal rivalry	363	0.21	0.06 (33%)	0.010
Sz vs. Ctrl	Distress	Paternal rivalry	363	0.21	0.05 (22%)	0.108

*Note*. *Ctrl* Siblings with a healthy brother or sister, *SZ* Siblings with a brother or sister experiencing schizophrenia

First, the predictor variable (e.g., having or not having a sibling with SZ) has to be significantly related to the outcome variable (e.g., emotional distress or self-esteem). Here, the predictor variable predicted emotional distress (*β* = 0.25, *p* < 0.05), but not self-esteem (*β* = 0.05, *p* > 0.05). Thus, self-esteem was not examined further.

Second, the predictor variable has to be related to the mediator variables (e.g., ASRQ-S indicators). This criterion was met for every ASRQ-S variable except for dominance and overall conflict (*β* = 0.10, *p* > 0.05 and *β* = 0.20, *p* = 0.05), explaining why neither of these variables was analyzed further.

Third, a mediation effect requires the mediator variable to be related to the outcome variable (e.g., emotional destress). As this condition was not met by the warmth dimension and three warmth subscales of the ASRQ-S (*β*s ranging from − 0.06 to − 0.03, *p* > 0.05), they were removed. By contrast, antagonism (*β* = 0.13, *p* < 0.01), quarreling (*β* = 0.14, *p* < 0.01), overall rivalry (*β* = 0.18, *p* < 0.001), maternal rivalry (*β* = 0.16, *p* < 0.01), and paternal rivalry (*β* = 0.12, *p* < 0.05) were all significantly related to emotional distress.

Finally, the initial effect of the predictor variable on the outcome variable must diminish when the mediator is entered as a second simultaneous predictor. Here, the effect of group (sibling with or without SZ) on emotional distress (*β* = 0.25, *p* < 0.05) fell to *β* = 0.22 (*p <* 0.05) when either antagonism or quarreling was entered in the regression analysis. These two mediation effects of *β* = 0.03 (11% mediation for antagonism and 10% for quarreling) were both statistically significant (*p* < 0.05).

The diminution of the effect of having a brother or sister with SZ on emotional distress was initially *β* = 0.21, if we removed missing data on rivalry-related variables. The effect of having a sibling with SZ fell to *β* = 0.12 (*ns*) when overall rivalry was included as a predictor of distress, and to *β* = 0.14, *ns* (or *β* = 0.17, *ns*) when maternal (or paternal) rivalry was included as a second predictor.

## Discussion

Ours was the first study to highlight differences in sibling relationships depending on whether a brother or sister had been diagnosed with SZ. These differences took the form of more negative experiences of sibling relations in the SZ group. In addition, the siblings of individuals with SZ experienced more emotional distress than the siblings drawn from the general population. Interestingly, our findings showed that this emotional distress was partly explained by antagonism, quarreling and rivalry in the relationship with the ill sibling.

### Warmth in sibling relationships

The siblings in our study who had a brother or sister with SZ felt less warmth in their sibling relationships than those who were drawn from the general population. Grieving for the relationship they used to enjoy with their sibling before he or she was diagnosed with SZ undoubtedly contributed to this feeling, as did the effort required to accept and adapt to this *new* brother or sister [58].

The siblings of individuals with SZ may sometimes experience fear. This fear is multifaceted, as it concerns not only their own mental health, but that of other family members, as well as the way other people view them and, of course, the suffering of their sibling [3, 4]. Bowman’s results suggested that a history of physical violence by brothers/sisters who have experienced an FEP significantly accounts for reduced warmth in sibling relationships [1]. Accordingly, our finding of a diminished feeling of warmth may be linked to a previous violent experience or assault.

In the present study, analysis of the ratings on the various ASRQ-S subscales revealed that the greatest intergroup difference concerned emotional support. According to Stålberg et al., healthy siblings find it hard to understand and anticipate the thoughts of their ill brother or sister [8]. This has a negative impact on communication and companionship within the dyad, and thus on the siblings’ willingness to be supportive. Some do nonetheless make several attempts to provide support, but often become worn down by successive relapses, and eventually give up [59].

### Conflict in sibling relationships

Our results also showed that the participants in the SZ sample reported more conflict in their sibling relationships than the participants drawn from the general population. The emotional burden, characterized by feelings of fear and impotence on the part of family members, can manifest itself as hostile and critical comments [60]. This hostility from close family and friends, reported in the literature in terms of *expressed emotion* [61, 62], substantially increases patients’ risk of relapse [63], and perhaps their suicidality. The effect is twofold, as quarrels within the relationship impact not only the ill brother or sister but also the healthy siblings, whose emotional distress increases, as the results of the present study interestingly suggest.

Moreover, ratings on the Antagonism subscale revealed that participants in the SZ sample expressed a greater need to differentiate themselves from their ill brother or sister, even though we observed that this search for differentiation formed part of their emotional distress. Fear of courtesy stigma [64] has been shown to heighten the desire of siblings to set themselves apart from a brother or sister living with a mental disorder.

As already mentioned, Dominance was the only ASRQ-S subscale on which the two groups did not differ. Having a sister or brother with a disability does not favor the emergence of any form of dominance. It is hard for healthy siblings to view a brother or sister with a disability as an equal with whom they could have a relationship of dominance without betraying family loyalty, which requires them to be protective [65].

### Rivalry in sibling relationships

Parents’ commitment to their mentally ill offspring can be a source of concern for their other children, who fear for their mental and physical wellbeing [4]. For these siblings, their most important role is to watch over and take care of their parents [3, 4]. This situation can make them feel lonely [3] and, in all probability, frustrated. The shifting positions and roles of the various family members generate comparisons and even criticisms among the healthy siblings, who start to resent their mentally ill brother or sister for monopolizing their parents’ attention and energy [3, 4]. Taken together, these different aspects can explain why we observed such an effect of rivalry on siblings’ emotional distress in the present study. Although authors have reported the opposite results for the siblings of children with developmental disorders, who actually express less rivalry than controls [66, 67], this may be because these young siblings feel guilty about competing with a brother or sister who has this type of disorder. So although they probably still feel rivalry, they express it less often [68]. Our results suggest that adults may feel more comfortable about expressing and admitting to this rivalry. Moreover, the later onset of SZ means that nonclinical siblings are suddenly forced to reassess their relationship with their mentally ill brother/sister. This late reassessment, coupled with a lack of understanding, may well generate a strong need for attention and support from their parents, such that the latter’s reduced accessibility actually heightens rivalry within the sibling relationship.

### Limitations and future research directions

The present study contributes to the literature on sibling relationships in adulthood and the specific features of these relationships in SZ. Indeed, our study was the first to compare siblings’ relationships with a brother or sister experiencing SZ with a matched group of siblings drawn from the general population. Nevertheless, it had several limitations.

First, our results cannot be generalized, as our sample did not have a balanced sex ratio and all the participants were French. It is not surprising that our sample was mainly made up of women, given that we collected our data via social media [69]. Moreover, female overrepresentation has been a recurring feature of research on adult sibships [16, 70]. It is unlikely that having a more balanced sex ratio would have changed our results, as Bowman et al. found no effect of sex on any of the three dimensions of sibling relationships (measured using the validated long version of the ASRQ) [1]. Our recruitment procedure may have introduced another sample bias. Participants were recruited either via social media (support groups for the relatives of individuals with mental disorders) or via the UNAFAM family organization. The advantage of this procedure is that it was not hospital-based, and therefore allowed brothers and sisters who had no links to psychiatric institutions to share some of their experiences as siblings. However, it meant that participants either had to be members of a charity or else had to belong to an online support group for family members, meaning that they did not represent all the brothers and sisters of individuals with SZ. In addition to these two criteria, despite its speed, autonomy and easy access, the use of the online procedure did require computer skills and a reasonable Internet connection, which may have put off some potential participants.

Second, there were biases in the way we matched the two groups. Whereas participants who had a brother or sister with SZ had to think about their relationship with him or her when filling in the questionnaire, unselected siblings could think about any brother or sister they chose. Although our participants were matched on more variables than in previous studies comparing groups on sibling relationships [42, 71, 72], we did not control for either age differences between the dyads, birth order, or the number of persons in each sibship, which are liable to influence dimensions of sibling relationships [50].

Finally, in the present study, we chose to investigate the sibling relationship on the three main dimensions that are classically studied in adult sibships: warmth, conflict, and rivalry. However, other aspects of the sibling relationship deserve to be explored in future studies. For instance, methodologies based on actor-partner interdependence models could be used to shed light on the reciprocal mechanisms involved in sibling relationships. In addition, it might be useful to compare the experiences of siblings who all have a brother or sister with SZ. Future research could also compare relationships involving a sibling with SZ and relationships involving a sibling with a different mental disability.

## Conclusion

To conclude, the quality of sibling relationships is a key area of research, owing to its impact on the continuing wellbeing and development of individuals throughout their lives [21]. The implementation of therapies that focus on strengthening the sibling relationships of individuals with SZ could help to protection their healthy siblings from emotional distress (e.g., depression and anxiety).

In order to strengthen relationships with siblings diagnosed with SZ, we first need to identify the variables that have the greatest influence on the quality of these relationships. In further studies, we therefore plan to identify the determinants of sibling relationships, both among sets of siblings drawn from the general population and among the siblings of individuals with SZ.

## Supplementary information

**Additional file 1.** Instructions and Basic Information.

## Data Availability

The datasets used and/or analyzed during the current study are available from the corresponding author on reasonable request.

## Abbreviations

ASRQ: Adult Sibling Relationship Questionnaire
ASRQ-S: Adult Sibling Relationship Questionnaire-Short Form
FEP: First episode of psychosis
HAD scale: Hospital Anxiety and Depression scale
*M*: Mean
*SD*: Standard deviation
SES: Rosenberg’s Self-Esteem Scale
SZ: Schizophrenia
UNAFAM: Union Nationale de Familles ou Amis de Personnes Malades et/ou Handicapées Psychiques (National Union of Families or Friends of Persons with Mental Disorders and/or Disabilities)

## Footnotes

1 National Union of Families or Friends of Persons with Mental Disorders and/or Disabilities.

2 A sparse Gaussian graphical model represents a network made up solely of the most important partial correlations between the examined variables, to facilitate the interpretability of the resulting structure. In the psychological network literature, this sparsity is frequently achieved using the least absolute shrinkage and selection operator (LASSO) regularization parameter, while the minimization of the extended Bayesian information criterion is the tuning parameter used by the LASSO to control the degree to which regularization is applied [55].
