# Supplementary material for: Comparison of relationships among French adult siblings with or without schizophrenia using the ASRQ-S: mediating effect on emotional distress
Source: BMC Psychiatry. 2020 Mar 13;20:122. doi: 10.1186/s12888-020-02510-6 (PMC7071713; doi:10.1186/s12888-020-02510-6)
Supplement: Supplementary file 1 — Additional file 1. Instructions and Basic Information. [file 12888_2020_2510_MOESM1_ESM.pdf]

Instructions and Basic Information

This questionnaire is concerned with your relationship with one of your siblings. Each question asks you to rate how much different behaviors and feelings occur in your relationship. Try and answer each question as quickly and accurately as you can. Try and answer the questions as your relationship is now, not how it was in the past, nor how you think it might be in the future. In the remainder of the questionnaire, whenever you see THIS SIBLING or YOUR SIBLING we are talking about the specific sibling you are completing the study about. We begin by asking you some general questions about your sibling and yourself. Please circle, check, or fill in the correct response.

1a) Your age: ________ 1b) This sibling's age: ________

2a) Your gender: Male Female 2b) This sibling's gender: Male Female

---

3a) Your birth order: 1 = firstborn, 2 = secondborn, 3 = thirdborn, 4 fourthborn, 5 = laterborn

---

3b) This sibling's birth order: 1 = firstborn, 2 = secondborn, 3 = thirdborn, 4 fourthborn, 5 = laterborn

---

How far does this sibling live from you? (circle the correct response)

1) same city
2) different city, less than 100 miles
3) between 100 & 200 miles
4) between 200 and 500 miles
5) between 500 and 1000 miles
6) more than 1,000 miles

How much do you and this sibling see each other?

[ ] 1 Hardly At All [ ] 2 A Little [ ] 3 Somewhat [ ] 4 Very Much [ ] 5 Extremely Much

---

How much does this sibling phone you?

[ ] 1 Hardly At All [ ] 2 A Little [ ] 3 Somewhat [ ] 4 Very Much [ ] 5 Extremely Much

---

How much do you phone this sibling?

[ ] 1 Hardly At All [ ] 2 A Little [ ] 3 Somewhat [ ] 4 Very Much [ ] 5 Extremely Much

---

How much do you and this sibling see each other for holidays and family gatherings?

[ ] 1 Hardly At All [ ] 2 A Little [ ] 3 Somewhat [ ] 4 Very Much [ ] 5 Extremely Much

---

What is your relationship to this sibling?

1) biological sibling
2) twin
3) step sibling
4) half sibling
5) other (please explain) ___________________________

Now we would like some information about your other siblings

DO NOT INCLUDE THIS SIBLING HERE

	Age	Gender	Relationship (bio, step, twin)		Age	Gender	Relationship (bio, step, twin)
Sib #1:	____	M F	______________	Sib #2:	____	M F	______________
Sib #3:	____	M F	______________	Sib #4:	____	M F	______________
Sib #5:	____	M F	______________	Sib #6:	____	M F	______________
Sib #7:	____	M F	______________	Sib #8:	____	M F	______________

Turn the page and begin the Adult Sibling Relationship Questionnaire

1) How much do you talk to this sibling about things that are important to you?
[ ] 1 Hardly Anything [ ] 2 A Little [ ] 3 Somewhat [ ] 4 Very Much [ ] 5 Extremely Much

2) How much does this sibling talk to you about things that are important to him or her?
[ ] 1 Hardly At All [ ] 2 A Little [ ] 3 Somewhat [ ] 4 Very Much [ ] 5 Extremely Much

3) How much do you and this sibling argue with each other?
[ ] 1 Hardly At All [ ] 2 A Little [ ] 3 Somewhat [ ] 4 Very Much [ ] 5 Extremely Much

4) How much do you irritate this sibling?
[ ] 1 Hardly At All [ ] 2 A Little [ ] 3 Somewhat [ ] 4 Very Much [ ] 5 Extremely Much

5) How much does this sibling irritate you?
[ ] 1 Hardly At All [ ] 2 A Little [ ] 3 Somewhat [ ] 4 Very Much [ ] 5 Extremely Much

6) Do you think your mother favors you or this sibling more?
[ ] 1 I am usually favored
[ ] 2 I am sometimes favored
[ ] 3 Neither of us is favored
[ ] 4 This sibling is sometimes favored
[ ] 5 This sibling is usually favored

7) Does this sibling think your mother favors him/her or you more?
[ ] 1 I am usually favored
[ ] 2 I am sometimes favored
[ ] 3 Neither of us is favored
[ ] 4 This sibling is sometimes favored
[ ] 5 This sibling is usually favored

8) How much does this sibling try to cheer you up when you are feeling down?
[ ] 1 Hardly At All [ ] 2 A Little [ ] 3 Somewhat [ ] 4 Very Much [ ] 5 Extremely Much

9) How much do you try to cheer this sibling up when he or she is feeling down?
[ ] 1 Hardly At All [ ] 2 A Little [ ] 3 Somewhat [ ] 4 Very Much [ ] 5 Extremely Much

10) How much do you dominate this sibling?
[ ] 1 Hardly At All [ ] 2 A Little [ ] 3 Somewhat [ ] 4 Very Much [ ] 5 Extremely Much

11) How much does this sibling dominate you?
[ ] 1 Hardly At All [ ] 2 A Little [ ] 3 Somewhat [ ] 4 Very Much [ ] 5 Extremely Much

12) Do you think your father favors you or this sibling more?
[ ] 1 I am usually favored
[ ] 2 I am sometimes favored
[ ] 3 Neither of us is favored
[ ] 4 This sibling is sometimes favored
[ ] 5 This sibling is usually favored

13) Does this sibling think your father favors him/her or you more?
[ ] 1 I am usually favored
[ ] 2 I am sometimes favored
[ ] 3 Neither of us is favored
[ ] 4 This sibling is sometimes favored
[ ] 5 This sibling is usually favored

14) How much does this sibling know about you?
[ ] 1 Hardly Anything [ ] 2 A Little [ ] 3 Somewhat [ ] 4 Very Much [ ] 5 Extremely Much

15) How much do you know about this sibling?
[ ] 1 Hardly Anything [ ] 2 A Little [ ] 3 Somewhat [ ] 4 Very Much [ ] 5 Extremely Much

16) How much do you discuss your feelings or personal issues with this sibling?
[ ] 1 Hardly At All [ ] 2 A Little [ ] 3 Somewhat [ ] 4 Very Much [ ] 5 Extremely Much

17) How much does this sibling discuss his or her feelings or personal issues with you?
[ ] 1 Hardly At All [ ] 2 A Little [ ] 3 Somewhat [ ] 4 Very Much [ ] 5 Extremely Much

18) How often does this sibling criticize you?
[ ] 1 Hardly At All [ ] 2 A Little [ ] 3 Somewhat [ ] 4 Very Much [ ] 5 Extremely Much

19) How often do you criticize this sibling?
[ ] 1 Hardly At All [ ] 2 A Little [ ] 3 Somewhat [ ] 4 Very Much [ ] 5 Extremely Much

20) How often does this sibling do things to make you mad?
[ ] 1 Hardly At All [ ] 2 A Little [ ] 3 Somewhat [ ] 4 Very Much [ ] 5 Extremely Much

21) How often do you do things to make this sibling mad?
[ ] 1 Hardly At All [ ] 2 A Little [ ] 3 Somewhat [ ] 4 Very Much [ ] 5 Extremely Much

22) Does this sibling think your mother supports him/her or you more?
[ ] 1 I usually get more support
[ ] 2 I sometimes get more support
[ ] 3 We are supported equally
[ ] 4 This sibling sometimes gets more support
[ ] 5 This sibling usually gets more support

23) Do you think your mother supports you or this sibling more?
[ ] 1 I usually get more support
[ ] 2 I sometimes get more support
[ ] 3 We are supported equally
[ ] 4 This sibling sometimes gets more support
[ ] 5 This sibling usually gets more support

24) How much can you count on this sibling to be supportive when you are feeling stressed?
[ ] 1 Hardly At All [ ] 2 A Little [ ] 3 Somewhat [ ] 4 Very Much [ ] 5 Extremely Much

25) How much can this sibling count on you to be supportive when he or she is feeling stressed?
[ ] 1 Hardly At All [ ] 2 A Little [ ] 3 Somewhat [ ] 4 Very Much [ ] 5 Extremely Much

26) How much is this sibling bossy with you?
[ ] 1 Hardly At All [ ] 2 A Little [ ] 3 Somewhat [ ] 4 Very Much [ ] 5 Extremely Much

27) How much are you bossy with this sibling?
[ ] 1 Hardly At All [ ] 2 A Little [ ] 3 Somewhat [ ] 4 Very Much [ ] 5 Extremely Much

28) Does this sibling think your father supports him/her or you more?
[ ] 1 I usually get more support
[ ] 2 I sometimes get more support
[ ] 3 We are supported equally
[ ] 4 This sibling sometimes gets more support
[ ] 5 This sibling usually gets more support

29) Do you think your father supports you or this sibling more?
[ ] 1 I usually get more support
[ ] 2 I sometimes get more support
[ ] 3 We are supported equally
[ ] 4 This sibling sometimes gets more support
[ ] 5 This sibling usually gets more support

30) How much do you know about this sibling's relationships?
[ ] 1 Hardly Anything [ ] 2 A Little [ ] 3 Somewhat [ ] 4 Very Much [ ] 5 Extremely Much

31) How much does this sibling know about your relationships?
[ ] 1 Hardly Anything [ ] 2 A Little [ ] 3 Somewhat [ ] 4 Very Much [ ] 5 Extremely Much

32) How much do you really understand this sibling?
[ ] 1 Hardly At All [ ] 2 A Little [ ] 3 Somewhat [ ] 4 Very Much [ ] 5 Extremely Much

33) How much does this sibling really understand you?
[ ] 1 Hardly At All [ ] 2 A Little [ ] 3 Somewhat [ ] 4 Very Much [ ] 5 Extremely Much

34) How much does this sibling disagree with you about things?
[ ] 1 Hardly At All [ ] 2 A Little [ ] 3 Somewhat [ ] 4 Very Much [ ] 5 Extremely Much

35) How much do you disagree with this sibling about things?
[ ] 1 Hardly At All [ ] 2 A Little [ ] 3 Somewhat [ ] 4 Very Much [ ] 5 Extremely Much

36) How much does this sibling put you down?
[ ] 1 Hardly At All [ ] 2 A Little [ ] 3 Somewhat [ ] 4 Very Much [ ] 5 Extremely Much

37) How much do you put this sibling down?
[ ] 1 Hardly At All [ ] 2 A Little [ ] 3 Somewhat [ ] 4 Very Much [ ] 5 Extremely Much

38) Does this sibling think your mother is closer to him/her or you?
[ ] 1 Our mother is usually closer to me
[ ] 2 Our mother is sometimes closer to me
[ ] 3 Our mother is equally close to both of us
[ ] 4 Our mother is sometimes closer to this sibling
[ ] 5 Our mother is usually closer to this sibling

39) Do you think your mother is closer to you or this sibling?
[ ] 1 Our mother is usually closer to me
[ ] 2 Our mother is sometimes closer to me
[ ] 3 Our mother is equally close to both of us
[ ] 4 Our mother is sometimes closer to this sibling
[ ] 5 Our mother is usually closer to this sibling

40) How much do you discuss important personal decisions with this sibling?
[ ] 1 Hardly At All [ ] 2 A Little [ ] 3 Somewhat [ ] 4 Very Much [ ] 5 Extremely Much

41) How much does this sibling discuss important personal decisions with you?
[ ] 1 Hardly At All [ ] 2 A Little [ ] 3 Somewhat [ ] 4 Very Much [ ] 5 Extremely Much

42) How much does this sibling act in superior ways to you?
[ ] 1 Hardly At All [ ] 2 A Little [ ] 3 Somewhat [ ] 4 Very Much [ ] 5 Extremely Much

43) How much do you act in superior ways to this sibling?
[ ] 1 Hardly At All [ ] 2 A Little [ ] 3 Somewhat [ ] 4 Very Much [ ] 5 Extremely Much

44) Does this sibling think your father is closer to him/her or you?
[ ] 1 Our father is usually closer to me
[ ] 2 Our father is sometimes closer to me
[ ] 3 Our father is equally close to both of us
[ ] 4 Our father is sometimes closer to this sibling
[ ] 5 Our father is usually closer to this sibling

45) Do you think your father is closer to you or this sibling?
[ ] 1 Our father is usually closer to me
[ ] 2 Our father is sometimes closer to me
[ ] 3 Our father is equally close to both of us
[ ] 4 Our father is sometimes closer to this sibling
[ ] 5 Our father is usually closer to this sibling

46) How much do you know about this sibling's ideas?
[ ] 1 Hardly At All [ ] 2 A Little [ ] 3 Somewhat [ ] 4 Very Much [ ] 5 Extremely Much

47) How much does this sibling know about your ideas?
[ ] 1 Hardly At All [ ] 2 A Little [ ] 3 Somewhat [ ] 4 Very Much [ ] 5 Extremely Much
